# Supplementary material for: What does it take to learn the rules of RNA base pairing? A lot less than you may think
Source: bioRxiv. 2025 Aug 2:2025.07.31.668042. Preprint. [Version 1] doi: 10.1101/2025.07.31.668042 (PMC12324431; doi:10.1101/2025.07.31.668042)
Supplement: Supplement 1 [file media-1.gz › supplemental_material/src/rscape_v2.6.0.tar.gz!/rscape_v2.6.0/lib/infernal/Manuscripts/inf-1-appnote/Proofs/btp157q.doc]

# AUTHOR/EDITOR QUERIES

| **Article ID** | **Section** | **Paragraph** | **Query** |
| --- | --- | --- | --- |
| bioinformatics-btp157 |  |  | Please provide the expansion of CYK. |
|  | References | Cole *et al.* (2009); Gardner *et al.* (2009) | Please update the references. |
|  | References | Eddy,S.R. (2003) | Please provide the last accessed date for the reference. |
|  | Funding |  | Please provide the expansion of “NIH NHGRI” in the funding section |
